# Theranostic applications of multifunctional carbon nanomaterials

**DOI:** 10.1002/viw.20220056

**Published:** 2023-03-03

**Authors:** Shima Masoudi Asil, Erick Damian Guerrero, Georgina Bugarini, Joshua Cayme, Nydia De Avila, Jaime Garcia, Adrian Hernandez, Julia Mecado, Yazeneth Madero, Frida Moncayo, Rosario Olmos, David Perches, Jacob Roman, Diana Salcido-Padilla, Efrain Sanchez, Christopher Trejo, Paulina Trevino, Md Nurunnabi, Mahesh Narayan

**Affiliations:** 1 Department of Environmental Science and Engineering, The University of Texas at El Paso, El Paso, Texas, USA; 2 Department of Biochemistry, Simmons Comprehensive Cancer Center, The University of Texas Southwestern Medical Center, Dallas, Texas, USA; 3 BUILDing SCHOLARS, Research Intensive Sequence (FYRIS) students, The University of Texas at El Paso, El Paso, Texas, USA; 4 Department of Pharmaceutical Sciences, School of Pharmacy, The University of Texas at El Paso, El Paso, Texas, USA; 5 Department of Chemistry and Biochemistry, The University of Texas at El Paso, El Paso, Texas, USA

**Keywords:** carbon nanotube, carbon quantum dots, diagnosis, fullerene, graphene, therapy

## Abstract

Nanobiotechnology is one of the leading research areas in biomedical science, developing rapidly worldwide. Among various types of nanoparticles, carbon nanomaterials (CNMs) have attracted a great deal of attention from the scientific community, especially with respect to their prospective application in the field of disease diagnosis and therapy. The unique features of these nanomaterials, including favorable size, high surface area, and electrical, structural, optical, and chemical properties, have provided an excellent opportunity for their utilization in theranostic systems. Carbon nanotubes, carbon quantum dots, graphene, and fullerene are the most employed CNMs in biomedical fields. They have been considered safe and efficient for non-invasive diagnostic techniques such as fluorescence imaging, magnetic resonance imaging, and biosensors. Various functionalized CNMs exhibit a great capacity to improve cell targeting of anti-cancer drugs. Due to their thermal properties, they have been extensively used in cancer photothermal and photodynamic therapy assisted by laser irradiation and CNMs. CNMs also can cross the blood-brain barrier and have the potential to treat various brain disorders, for instance, neurodegenerative diseases, by removing amyloid fibrils. This review has summarized and emphasized on biomedical application of CNMs and their recent advances in diagnosis and therapy.

## 1 | INTRODUCTION

The convergence of nano and biotechnology enables scientific and technical knowledge to improve human well-being. Nanotechnology products have become progressively applicable in biomedicine and have led to the emergence of hybrid science-denominated nanobiotechnology.^1^ Nanoscale materials incorporate very well into biomedical devices because most biological techniques are designed based on nanosized systems. The nanoparticles offer several advantages compared to larger particles, such as an increased surface-to-volume ratio, which provides a higher density of active sites for biological reactions and makes them suitable for biomedical purposes. The most commonly used materials to develop these nanotechnology products are inorganic and metal nanoparticles, metallic surfaces, liposomes, and carbon nanomaterials (CNMs).^2^

For over 6000 years, carbon was mainly used to reduce metal oxides.^3^ Until the 1980s, graphite, diamond, and amorphous carbon were the only three known carbon allotropes. Since the discovery of fullerenes in 1985, CNMs, known as carbonaceous materials with low dimensions, has attracted considerable interest among scientists. Over the past 30 years, different carbon nanostructures have been developed, namely graphene, graphene oxide (GO), fullerenes, carbon nanotubes (CNTs), nanowires, nanoribbons, and carbon quantum dots (CQDs).^4^ Owing to unique structural, chemical, electronic, and optical properties, they have been developed with multifunctional applications in multiple fields of science. In recent years, CNMs have been developed for various applications in electronics, optics, biomedical engineering, bioimaging, drug delivery, medical devices, tissue engineering, medical implants, and biosensors; they have found an exceptional niche in biomedical science.^5–7^ The suitable sizes of CNMs have made these nanocapsules and nano-carriers favorable materials to load and deliver drugs and genes to specific targets in vivo. There are remarkable advancements in the application of CNMs in regenerative medicine, tissue engineering, gene transferring, and drug delivery.^8^

**FIGURE 1:**
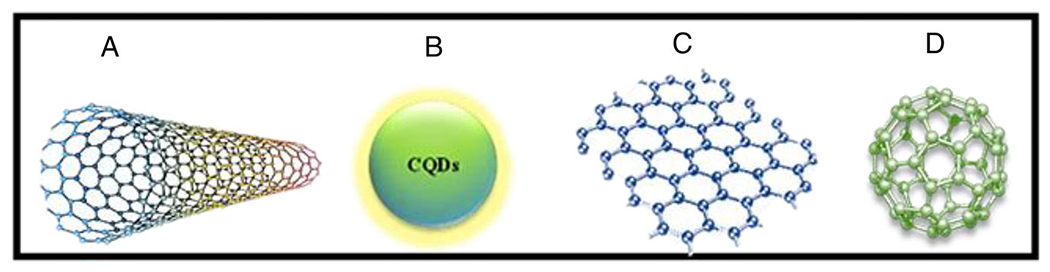
Carbon-based nanomaterials: (A) carbon nanotubes, (B) Carbon quantum dots, (C) graphene, and (D) fullerene.

Here, we will introduce the different types of CNMs, including fullerene, CNTs, CQDs, and graphene (Figure 1). Then will address recent advances in the multifunctional applications of various CNMs in biomedical fields such as therapy and diagnosis.

## 2 | CARBON NANOMATERIALS

### 2.1 | Carbon nanotubes

CNTs are tube-shaped materials that contain one or many concentric graphite-like layers with diameters in the range of 1 to 100 nm, while the length can be up to several centimeters. The CNTs can be defined as graphite sheets rolled up into cylindrical forms and are commonly synthesized using three different methods, including arc discharge, laser ablation, and chemical vapor deposition.^9^ Two types of CNTs are categorized according to their carbon layer numbers. Single-walled CNTs (SWCNTs) comprise a single graphene layer with diameters ranging between 0.4 and 2 nm and usually develop as hexagonal-packed bundles. Multi-walled CNTs (MWCNTs) include two to several cylinders, each made of graphene sheets, with diameters varying from 1 to 3 nm.^9^ Because of the large p–p bonds covered on the CNTs surface, they can come over various biological barriers within the body and improve drug delivery efficiency.^10^ They have attracted huge attention in biomedicine research as advanced nano-vectors deliver various therapeutic molecules through p–p interactions. CNTs possess unique properties that equip them for multiple biomedicine purposes.^11^ However, the raw CNTs are highly hydrophobic in character, and therefore, their surface modification and functionalization to disperse them into aqueous solutions is a key step for their biomedical applications. The CNTs modification strategies are including non-covalent and covalent interactions which make them suitable candidates for various biomedical applications.^12^

### 2.2 | Carbon QDs

Since their discovery, QDs have become beneficial materials due to their great features, such as electrical, antimicrobial, and optical properties.^13,14^ The dots are known as “quantum” because their size presents quantum properties that can alter the energy levels of electrons in particles. CQDs, a new member of CNMs with zero dimension, are an active source of active oxygen and were first discovered during the purification of SWCNTs from arc-discharged soot in 2004.^15^ CQDs are synthesized through two methods: the top-down route and the bottom-up route. CQDs typically have quasi-spherical shapes with amorphous nanocrystalline cores, which mainly form graphitic, turbostratic carbon (sp_2_ carbon), or graphene and GO sheets integrated with diamond-like sp_3_ hybridized carbon insertions. As a new type of the nanocarbon family, CQDs are intriguing QDs for numerous applications in cancer therapy, biosensing, bioimaging, chemical sensing, drug delivery, photodynamic therapy (PDT), electrocatalysis, and photocatalysis.^16,17^ CQDs exhibit less cytotoxicity than their traditional QD analogs as the absence of heavy metals in their structure.^18,19^ Their sizable surface ensures their binding to many different compounds, which is an attractive and valuable property in a drug delivery system, that is, their potential to bind to several anticancer drug molecules.^20^ In particular, fluorescent CQDs of size less than 10 nm have attracted considerable research owing to their favorable luminescent properties, high chemical stability, facile surface functionalization, low toxicity, and biocompatibility.^21^

### 2.3 | Graphene

Graphene is the prime carbon-based two-dimensional atomic crystal and has garnered extensive attention since its discovery by Geim and co-workers in 2004.^22^ It is a single-atom, two-dimensional carbon sheet with hexagonally ordered sp_2_ hybridized carbon atoms, and its isolation from crystalline graphite and characterization led to the Nobel Prize in 2010.^23^ A variety of functionalized forms of graphene-based nanomaterials have been developed and well-studied. The graphene family varies based on the wall number (single/multi-walled), surface chemistry, dimensions (length and diameter), layer number, quality of sheets, and purity. Various types include monolayer graphene (a single-layer thickness), few-layer graphene (contains 2–10 flake-like stacks of graphene layers), ultra-thin graphite (with a thickness greater than 3–5 nm but less than 100 nm), GO (single-atom-thick carbon sheets with carboxylate groups on the surface), and reduced GO (rGO) (rGO under conditions such as high-temperature thermal treatment or treated with reducing agents).^24^ These novel nanomaterials have unique properties, such as large surface area, versatile surface, improved biocompatibility, photothermal function, pH sensitivity, and so forth.^25,26^ They can also be functionalized with various polymers through covalent or non-covalent bonds to minimize their toxicity and develop their specific/selective interactions with the cells.^27,28^ Graphene and its nanocomposites have recently been widely employed in biomedicine for bioimaging, biosensing, cancer therapy, drug/gene delivery, and tissue engineering.^26,29–37^

### 2.4 | Fullerene

Carbon has various allotropes like diamond, graphite, and so forth. The third allotropic form of carbon is fullerene.^38^ The development of research on this carbon allotrope and its various derivatives began three decades ago with Kroto and his team, who discovered fullerene as a novel allotropic form of carbon. Buckminsterfullerene (C60) is a spherical icosahedron where 60 carbon atoms form pentagons through C5–C5 single bonds and hexagons with C5–C6 double bonds. The diameter of a C60 fullerene molecule is 0.7 nm; therefore, it is an important member of the CNMs family.^39^ Since discovering C60 fullerene, it has attracted great interest in medicinal chemistry due to its high potential as a biologically active compound. Due to unique geometrical shapes, novel photophysical characteristics, and high efficiency in radical scavenging, fullerenes are considerably employed in broad biological applications.^40^ Buckminster fullerenes are powerful “radical sponges” because of their ability to neutralize multiple radicals per each fullerene molecule. However, a biological system can only be treated with this radical scavenging function when the fullerene’s water solubility is improved.^41^ The presence of *π* electron moiety in fullerenes causes improving intermolecular interactions; however, it also makes them insoluble in most common solvents. This negative feature of these molecules for use in medicinal chemistry, low solubility in polar solvents, and formation of aggregates in aqueous solutions can be fixed through chemical or supramolecular approaches.^42^ Functionalization of fullerenes through their integration into the ionic and nonionic groups enhances their solubility in polar solvents and easily overcomes the limitations caused by C60.^43,44^ By using radical polymerization with vinylpyrrolidone, Jiang et al. developed a nanoball form of fullerene derivatives with high water solubility.^45^ C60 and its derivatives have been considered potent carriers for drug delivery on account of their great fluorescence properties and low toxicity. The C60 has been widely employed in PDT.^46^ Moreover, C60 fullerene has been considered a potent therapeutic agent for the treatment of diseases such as cancer, diabetes, Parkinson’s, and Alzheimer’s.^47^

## 3 | DIAGNOSIS APPLICATIONS

Rapid diagnosis and precise detection of the early stages of a disease is the most effective approach to accelerate treatment processes or enhance patients’ survival. Continuous screening is also required for clinical treatment and monitoring of disease recurrence post-therapy.^48^ Therefore, developing high-throughput, ultra-sensitive, and cost-effective modern diagnostic techniques has become a critical research field. The engagement of new complex nanomaterials in diagnostic systems and the feasibility of their processing and modification have become a challenging but necessary research topics. Recent research trends aim to discover and explore nanomaterial’s potential in biomedical applications, and in particular, as a rising star, CNMs have drastically attracted considerable interest in diagnosis techniques.^49^ Image-guided carbonaceous nanomaterials can assess the therapeutic efficacy of specific therapy by tracking the fluctuation of target proteins/receptors levels.^50^ More essentially, the diverse abilities of nanomaterials provide opportunities to use them as multifunctional tools for theranostic, a combination of therapy and diagnosis. Hence, nanomaterials can be applied as a single system to localize and treat tumors, monitor the treatment progress, and report the therapeutic effects.^51^ Here, we will review the recent developments in diagnosis applications, including bioimaging and biosensor techniques using CNMs. The diagnosis applications of carbon-based nanomaterial are summarized in Table 1.

### 3.1 | Bioimaging

Biomedical imaging is a critical technique in cancer diagnosis, intraoperative guidance, and post-surgery inspection. In the past few decades, imaging technologies have been considerably developed and applied in clinical tests, such as MRI, fluorescence imaging, photoacoustic imaging (PAI), computed tomography (CT), ultrasound, single-photon emission CT, and positron emission tomography.

#### 3.1.1 | Magnetic resonance imaging

MRI is a non-invasive imaging technique that can provide original 3D cross-section images without ionizing radiation damage to the human body and with high resolution to various tissues.^77^ Recently, CNMs have been effectively established as new MRI contrast agents.^78^

**FIGURE 2:**
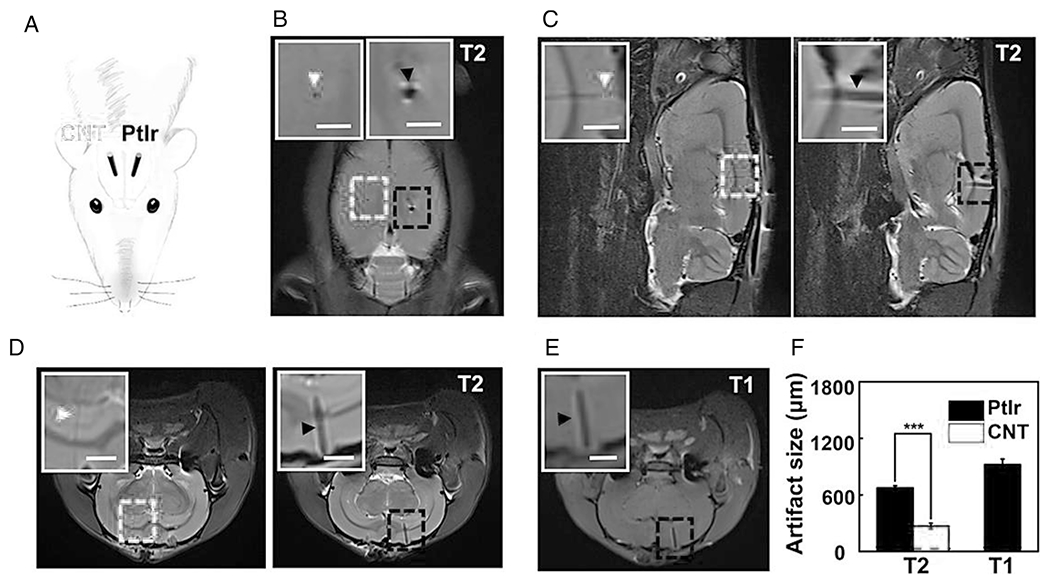
In vivo magnetic resonance imaging (MRI) artifact assessment using carbon nanotubes (CNTs). (A) Schematic diagram of a rat implanted contralaterally with a CNT fiber and PtIr microwire used for MRI studies. (B–D) Horizontal (B), sagittal (C), and coronal (D) sections of the T2-weighted images of a rat implanted contralaterally with a Parylene-C-insulated CNT fiber and PtIr microwire. The insets are zoomed-in photographs of the red or blue boxes. Scale bar, 1.5 mm. The CNT fiber and PtIr microwire are in different planes in the sagittal and coronal images. (E) Coronal section of the T1-weighted image of the rat implanted contralaterally with a Parylene-C-insulated CNT fiber and PtIr microwire. The red and blue dashed boxes/arrows mark the CNT fiber and PtIr microwire, respectively. (F) MRI artifact size of the Parylene-C-insulated CNT fibers and PtIr microwires. The black dashed line denotes their actual size. CNT fibers are invisible in T1-weighted images. Error bars denote SEM, *n* = 5. ***p < 0.001; one-way analysis of variance (ANOVA). All CNT fibers and PtIr microwires used here are insulated with ~2.5 *μ*m Parylene-C. Reproduced with permissions.^55^ Copyright ^©^ 2019, American Chemical Society.

Carbon nanotubes, due to their ultrahigh surface area, excellent mechanical strength, and rich electronic polyaromatic structure, are potential tools for diagnosis techniques such as MRI.^79^ The modification of CNTs allows us to conjugate magnetic contrast agents and drugs to construct multifunctional and hybrid nanomaterials for magnetic resonance imaging (MRI). One of the main issues with common imaging contrast agents is their limitation to cross the cell membrane. The contrast agents can be delivered intracellularly by linking onto the CNTs and their penetration can be facilitated through the CNTs’ ability to target the cells. Moreover, CNTs cavities and their large specific surface can enhance their loading capacity to hold metal ions or magnetic iron oxide nanoparticles (IONP), the main current MRI contrast agents. SWCNT-loaded gadolinium-diethylenetriamine pentaacetic acid (Gd-DTPA) (MRI contrast agent) was successfully employed to target the tumor site and transport Gd-DTPA into MCF-7 cells and showed the potential to provide high-resolution MR images.^52^ Gd^3+^, due to having seven unpaired electrons, exhibits high magnetic properties and is used in MRI contrast compounds. Gadonanotubes are new nanohybrid tools for isolating Gd^3+^ ions by encapsulating them within (or upon) ultrashort carbon nanotube capsules, which can increase their dispersibility in water. This nanoplatform system can be used in labeling cells for MRI and phantom imaging experiments.^53^ Modification of CNTs with metals is another strategy to improve their efficiency as MRI agents. Iron-containing MWCNTs are bifunctional nanoparticles capable of being localized in tumor sites by MRI, and to reduce iron toxicity, they can be entrapped within the MWCNT rather than placed on the surface.^54^ Soft and MRI-compatible neural electrodes were developed using 5–20 *μ*m CNT fibers. These nanocarbon-based fibers have great interfacial electrochemical properties and efficiently enhance MRI scanning quality. This soft CNT fiber electrodes improve specific targeting of the brain with high-quality single-unit neural signals. Compared to original stiff metals, these soft electrodes can significantly reduce brain inflammatory responses (Figure 2).^55^

**FIGURE 3:**
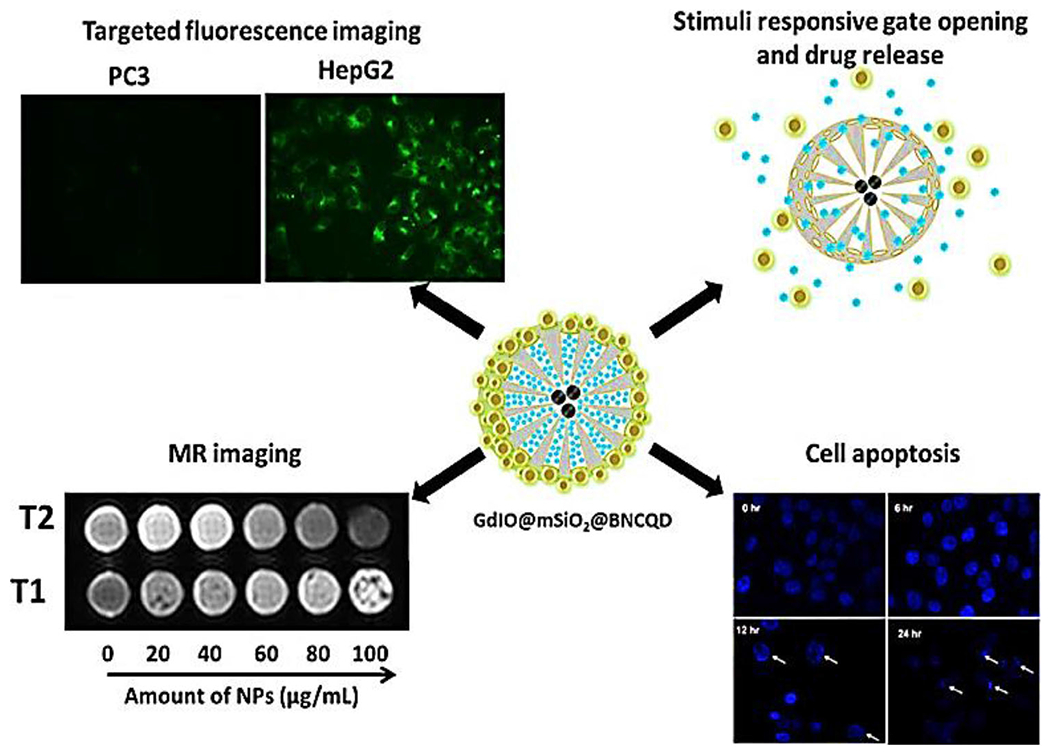
A novel multifunctional hybrid nanoparticle platform fabricated from magnetic mesoporous silica gated with doped carbon dot for site-specific drug delivery, fluorescence, and magnetic resonance (MR) imaging. Reproduced with permissions.^57^ Copyright^©^ 2018, American Chemical Society.

The CQDs can become magnetic for MRI purposes while simultaneously having the advantage of drug delivery and fluorescence imaging. The MRI fused with fluorescence imaging properties of CQDs would have improved the tissue penetration efficiency, the spatial resolution of MRI, and the convenient microscopic tissue evaluation of the fluorescent imaging. A multifunctional platform made of Fe_3_O_4_@CQDs coated SWNTs with magnetofluorescent properties was successfully fabricated and used as a dual-targeting agent for modal fluorescence/MRI. This complex had multifunctional advantages, serving as an efficient probe for MRI and fluorescent bioimaging, a drug loading carrier, a near-infrared (NIR) photothermal heater, and a NIR reactive oxygen species (ROS) generator, therefore acting on simultaneous imaging and cancer therapy.^56^ Another highly efficient nanohybrid complex was developed of magnetic gadolinium oxide–iron oxide core, mesoporous silica shell assembled with boronic acid functionalized highly luminescent carbon quantum dot. This smart theranostic complex is pH-sensitive for controlled drug delivery and owns considerable good relaxivity of the magnetic core and great fluorescence properties of the doped carbon quantum dot; it has promising potential to be developed as an MRI contrast agent and/or fluorescence imaging probe (Figure 3).^57^

The most dominant application of fullerene for cancer imaging is for MRI.^80^ Gadofullerene encapsulated redox nanoparticles (Gd_3_NPs) can serve as an excellent MRI contrast agent with high-resolution contrast between tumor tissues and adjacent muscle tissues with extended blood circulation and without significant adverse effects.^58^ To diagnose and treat Alzheimer’s disease, C60 was conjugated to an up-conversion nanoparticle system, and the nano-based platform could simultaneously protect neurons from A*β* aggregation and serve as an MRI agent. This C60-conjugated system was performed successfully for MRI imaging and could be visualized and colocalized with the anti-A*β* antibodies in neural PC12-A*β* cells, which is beneficial for image-guided therapy.^59^ However, the original gadofullerenes are insoluble in water; therefore, they need to be functionalized with hydrophilic groups for use as MRI contrast agents. The modification of gadofullerene with GO could enhance the interaction of Gd^3+^ ions of carbon nanohybrids with the surrounding water molecules.

Moreover, the two-dimensional planar structure of GO and the many hydrophilic groups on the GO surface both improve the relaxivity of gadofullerene conjugated with GO nanohybrids. Magnetic fluorescent graphene functionalized with silicon napthalocyanine bis developed as a theranostic nanocarrier and, due to its superparamagnetic nature, could serve as an effective MRI contrast reagent.^60,81^ The complex of GO modified with IONP was fabricated to detect pancreatic cancer metastasis of regional lymph nodes (RLN). They exhibited a strong ability for dual-modality mapping of regional lymphatic system MRI. Moreover, the dark color of the probe provided essential information for the surgeon in preparing the preoperative map before the operation and intraoperatively detecting RLN from peripheral tissue.^61^

#### 3.1.2 | Fluorescence imaging

Fluorescence imaging provides higher temporal and spatial resolution than other imaging modalities. In this technique, a beam of light is used to illuminate the specimen, and a camera collects another light radiation with a longer wavelength emitted from the specimen. This method offers images within milliseconds, with a high resolution of up to tens of nanometers.^50^ Some CNMs can produce fluorescence in the visible and infrared spectra for fluorescence imaging.^82^

The intrinsic fluorescence in the second NIR region (1100–1400 nm, NIRII) from CNTs can be readily employed for in vivo tumor detection.^83^ These biocompatible, functionalized SWNTs can circulate through organs such as lungs and kidneys for several seconds after injection and in the liver and spleen for slightly longer periods. Therefore, they are potent, powerful tools for high-resolution optical imaging in disease diagnosis.^62^ Single-chirality DNA-encapsulated SWNTs have been examined for their short- and long-term biodistribution and biocompatibility upon intravenous administration into the mice’s liver. Imaging the organs using NIR hyperspectral microscopy localized the nanotubes in other organs such as the spleen, lungs, kidney, and hearts for up to five months.^62^ Functionalized SWNTs with phospholipids polymers are biologically safe and long-circulating CNMs with intrinsic NIR photoluminescence (NIR PL). Intrinsic fluorescence of functionalized SWNTs facilitates video-rate tumors’ imaging in the second NIR (NIR-II, 1.1–1.4 *μ*m) window. The captured images demonstrated improving tumor contrast up to 72 h post-injection, which allows their precise identification in the target site. Moreover, a great extent of colocalization and stable PL of SWNTs was visualized using the 3D reconstruction of the SWNTs distribution inside the tumor.^63^ The SWNTs can also be assembled into the engineered phage and act as a fluorescence imaging probe for specific localization and therapy monitoring of hard-to-detect regions.^84^

**FIGURE 4:**
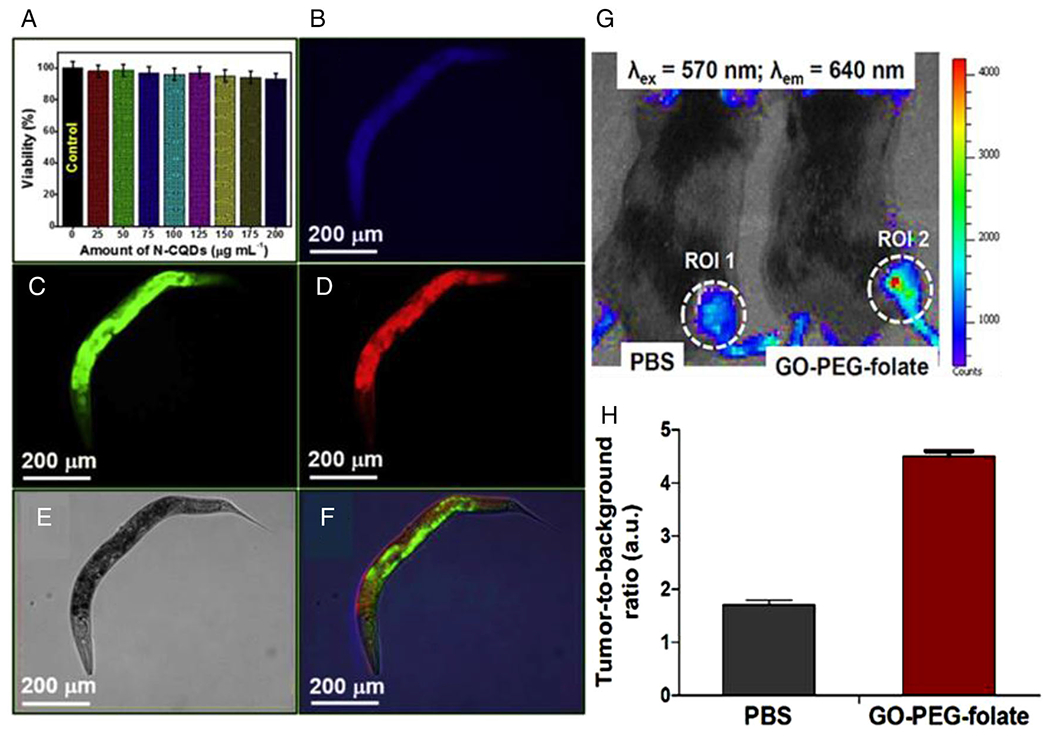
Fluorescence bioimaging of carbon quantum dots (CQDs). (A) Toxicity assay of *C. elegans* incubation with nitrogen-doped CQDs (N-CQDs) at different concentrations prepared from *P. acidus* fruit. Multicolor in vivo model *C. elegans* incubation with N-CQDs prepared from *P. acidus* fruit imaging under the excitation of (B) 400 nm, (C) 470 nm, (D) 550 nm, (E) bright field (BF), and (F) merge (overlap). Live *C. elegans* were immobilized using 0.05% sodium azide (NaN3) for imaging under fluorescence filters (G) In vivo fluorescence imaging of graphene oxide-polyethyleneglycol (GO-PEG)-folate. Near-infrared (NIR) fluorescence images from phosphate-buffered saline (PBS) (left) and GO-PEG-folate (right) mice at 24 h after intravenous injection. (H) Tumor-to-background ratio (TBR) integrated from the NIR fluorescence images for both PBS and GO-PEG-folate, respectively. Reproduced with permissions.^64,68^ Copyright^©^ 2019 Elsevier B.V., and ^©^2016 Elsevier Ltd.

CQDs with low toxicity, wavelength-dependent luminescence, low photo-bleaching, and photo-stability are developing as great candidates for fluorescence imaging probes.^85^ Nitrogen-doped CQDs (N-CQDs) are potent probes for multicolor imaging in *Caenorhabditis elegans* (*C. elegans*) as an in vivo model. These fluorescent CQDs were successfully employed for high-contrast imaging to distinguish living and dead *C. elegans* (Figure 4A–F).^64^ The CQDs can serve as a NIR light-triggered photoacoustic (PA) imaging tool in vivo. C-dots with red emission (from 500 to 800 nm with a peak at 640 nm) from a polymer, polythiophene phenylpropionic acid, were used as multimodal fluorescent in HeLa tumor-bearing nude mice. The mice in the experimental groups were intravenously injected with CQDs and then irradiated with a 671 nm laser and 2–5 h post-injection. The images showed that the CQD treatment caused a quick increase in their accumulation in the tumor area through the enhanced permeability and retention effect.^65^ The surface passivation of CQDs can alter their optical properties in fluorescence imaging techniques.^86^ The high fluorescence quantum yield CQDs synthesized from L-cysteine and citric acid were successfully employed for the in vitro imaging of gastric carcinoma cells. Their excellent luminescence properties are ascribed to amidogens and sulfhydryl groups of synthesized CQDs. Moreover, in the fluorescence images, the high absorbance rate of the CQDs by living cells was observed.^66^ A multifunctional nanosystem fabricated using sulforaphane-conjugated C-dots has offered an effective fluorescence probe to target cancer cells with biological and chemical advantages, mainly including early detection and control of cancer, high biocompatibility, nontoxicity, versatile surface modification, green synthetic method, and optical stability.^67^

The fluorescence imaging potential of GO has also been evaluated using a GO-PEG-folate complex. This compound was used in vivo for the localization of cancer cells and was intravenously injected into melanoma cancer cell tumor-bearing mice. GO-PEG-folate could exhibit single-photon excitation wavelength-dependent PL while administered under very low laser doses (5–10 mW/cm^2^), demonstrating its potential to exert fluorescent imaging (Figure 4G,H).^68^

#### 3.1.3 | Photoacoustic imaging

PAI is a biomedical imaging technique widely used for monitoring biological responses in small animals. This imaging technique has the unique advantages of both optical and ultrasound imaging and provides important molecular information from the multispectral PA responses of biological samples. The basics of PAI’s function arise from the PA effect, which transforms energy from light to acoustic waves. In the PAI technique, the pulsed laser applied to the biological tissue is absorbed by the chromophores and covert to heat energy quickly. The fast conversion of energy to heat makes the acoustic waves (PA waves), and then the conventional ultrasound (US) transducers detect the multiplied PA waves, which are then converted to high-US resolution digital signals for imaging in deep tissue. CT is one of the major branches of PAI (PACT) configuration. The main privilege of PACT is covering a larger area with fast image acquisition, which provides a wider and deeper imaging surface in the transversal plane.

The unique characteristics of CNMs, including functionalization with drugs/inorganic/organic materials, surface modification, and fabrication method, improves both PA signal intensity and theragnosis functionality.

Ultraselective SWCNs combined with PAI can detect inflammatory Ly-6Chi monocytes and foamy macrophages, as an indication of early-stage high-risk atherosclerotic plaques. PAI combination with intravenously injected CNTs improves inflamed atherosclerotic plaque detection by a ≈6-fold greater signal.^87^ Zeolitic imidazolate-derived carbon nanoparticles (ZCNs) as photothermal agents and photosensitizers (PSs) can produce heat and induce ROS to treat cancer. The therapeutic effect of ZCNs guided by PA imaging is investigated in eliminating tumors in a small animal model with minimal side effects and high efficacy.^88^

The nanoplatform composed of mesoporous carbon nanoparticles was employed as NIR-responsive chemotherapeutic drug doxorubicin (DOX) carrier. NIR light was observed by this carbon-based nanoplatform and converted into sufficient heat to induce drug release in the acidic microenvironment of the tumor. More importantly, the nanoplatform had higher cancer therapy efficacies under the guidance of PAI in combination with chemotherapy, photothermal therapy (PTT), and gas therapy.^89^

In an effort to employ CNMs for cancer therapy purposes, supra-carbon nanodots with high photothermal conversion efficiency were used as high NIR light absorbent, excellent photothermal, and PAI agents. These CNMs could reach the tumor site through blood circulation after intravenous injection.^90^ Recently a nanotheranostic platform composed of ammonic borane loaded into hollow carbon NPs was developed for imaging targets in the NIR-II bio-range (1000–1700 nm). This nanoplatform exhibited high intratumoral accumulation and PA-guided precise NIR-II PTT.^91^

Recently, PACT has attracted more attention as a hybrid imaging technique in preclinical and clinical research that provides simultaneous light absorption measurements and acoustic detection. The endogenous or exogenous contrast agents that absorb optical signals are required for biomedical PACT imaging.^92^ Carbon nanotubes have been used as nontoxic exogenous contrast boosters to enhance the sensitivity and contrast of tumor imaging.^93^ Radiolabelled f-MWNTs were successfully injected intravenously into the brain parenchyma and capillaries as nanocarriers for PACT imaging, and have been found as potent neurological therapeutics.^94^

### 3.2 | Biosensors

In contrast to traditional methods, which should be operated in clinics and laboratories by expert personnel and are laborious, biosensors can diagnose precisely and rapidly at the point of care for patients.^95^ Sensors have been particularly aimed at focusing on miniaturization using novel materials for their fabrication.

The attractive features of nanomaterials are demonstrated in their multiple benefits, including tiny size and an extreme increase in the surface area, rendering them a huge potential for versatile applications. Carbon nanomaterials provide attractive opportunities for improving biosensor performances among various nanoparticles due to their exceptional electric and mechanical properties, high specific surface area, and biocompatibility.^95–96^ The integration of CNMs with biosensor platforms is now developing rapidly in biosensor design. The most widely used CNMs to date are nanotubes (SWCNTs and MWCNTs), CQD, graphene, GO, rGO, and fullerene (C60), which emerge as novel materials for sensor construction.^97^

The number of circulating tumor cells (CTCs) in peripheral blood is an important indicator for the early diagnosis and metastasis rating of tumor patients. Therefore, there is a crucial need to trap and detect the low number of CTCs through precise and effective assays. Based on the peroxidase-like activity of SWCNTs and the effect of DNA sequences on this activity, a colorimetric probe was developed to detect the rare number of CTCs in PBS and lysed blood.^69^ The CNTs can be modified to develop biosensors to detect biomarkers involved in cancer. A nanocomposite was fabricated using peroxidase-like graphene QDs (GQDs) and SWCNTs and employed as an enzyme-free electrochemical immunosensor to detect carcinoembryonic antigen (CEA).^70^

**FIGURE 5:**
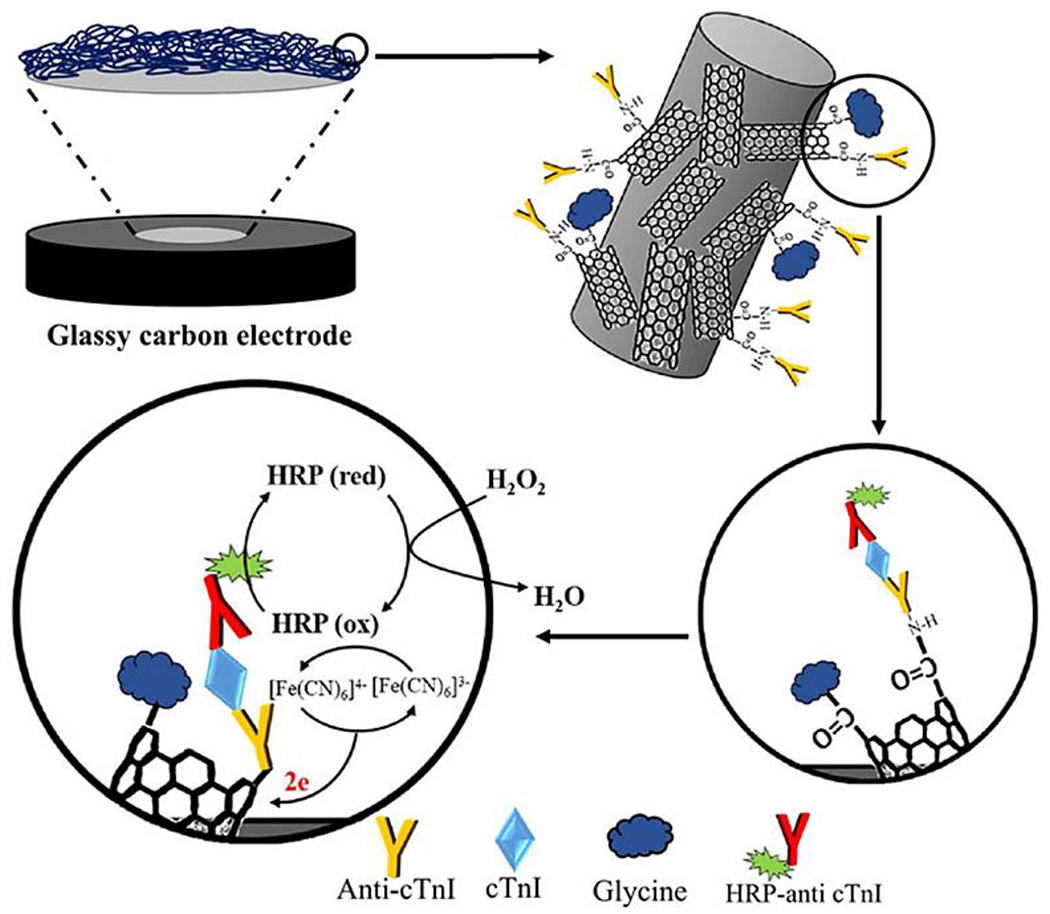
Schematic view of the stepwise fabrication of immunosensor and the mechanism of detection. Reproduced with permission.^71^ Copyright^©^ 2018 Elsevier B.V.

Moreover, to amplify the electrodes’ signals, the rGO and gold nanoparticles were applied because of their ultrahigh-specific surface area and excellent conductivity. This immunosensor exhibited good specificity and sensitivity toward the detection of CEA with a low detection limit.^98^ A sandwich-type nanoplatform immunosensor was fabricated using carboxylated MWCNT (CMWCNT)-embedded whiskered nanofibers for the detection of cardiac Troponin I (cTnI) as a cardiovascular disease biomarker. The CMWCNTs could successfully anchor and stabilize within the nanofibers with significant electrochemical repeatability. In this sensor, cTnI and horseradish peroxidase-conjugated anti-cTnI (HRP-anti-cTnI) formed a sandwich-type immuno-complex. This nano-based immunosensor provided a wide detection range for cTnI for clinical tests with high sensitivity, which make it an ideal nanostructure immunosensor tool for point-of-care testing (Figure 5).^71^

**FIGURE 6:**
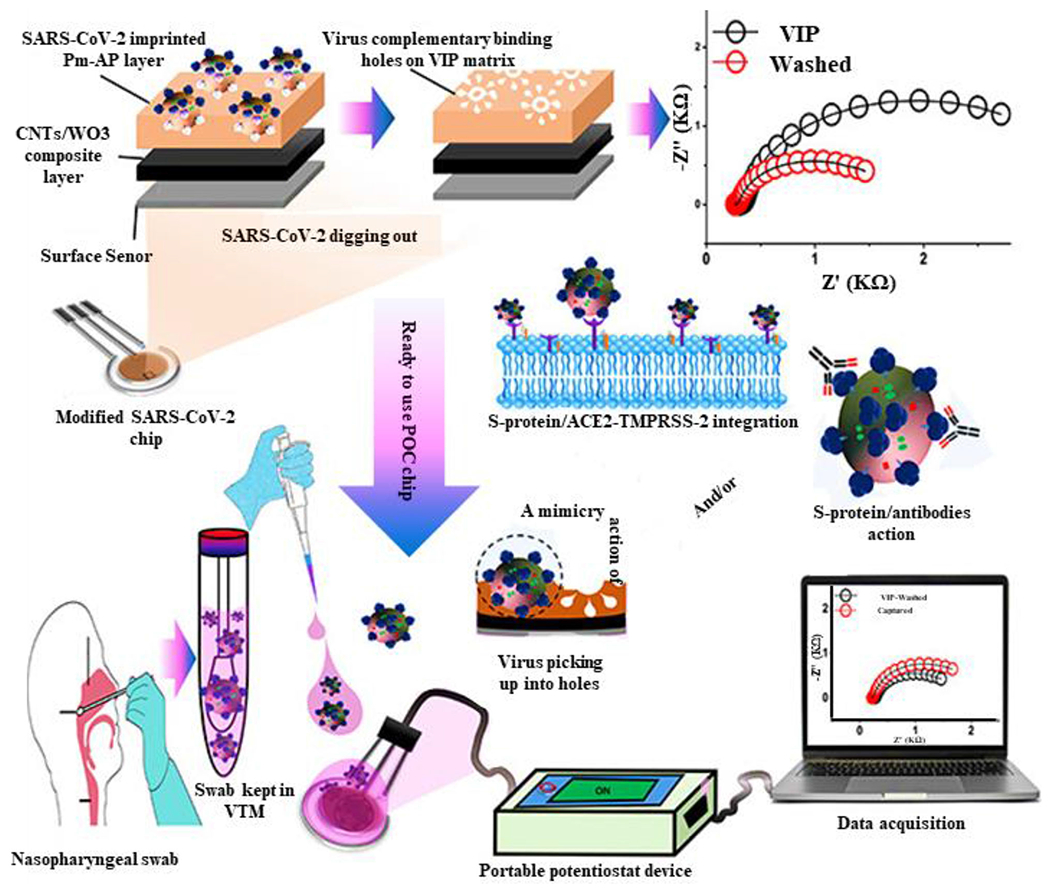
Carbon nanotube (CNT)-based severe acute respiratory syndrome coronavirus 2 (SARS-CoV-2) biosensor. Steps of fabrication of SARS-CoV-2 variable importance of projection (VIP) biosensor using CNTs combined with tungsten oxide (CNTs/WO3)-screen printed electrodes for imprinting the complete virus particles and testing the designed sensor for clinical sample analysis. Reproduced with permission.^73^ Copyright^©^ 2021 American Chemical Society.

MicroRNAs provide reliable information as biomarkers for the early diagnosis of cancer. By a combination of electrochemistry and nanotechnology, an electrochemical biosensor was designed to detect miR-21. This nano-genosensor was fabricated using Au-NPs, fluorine-doped tin oxide electrode grafted on the SWCNTs. This nano-genosensor exhibited excellent efficiency in the detection of miR-21 amplicon as a biomarker for the early detection of prostate cancer.^72^ Upon the emergence of the recent global issue, the SARS-CoV-2 pandemic, the necessity of developing rapid and accurate diagnostic tools has increased. CNTs combined with tungsten oxide (CNTs/WO3) have been used for developing electrodes of an electrochemical sensor of the virus-imprinted chip to detect the SARS-CoV-2 virus particles. This CNTs/WO3 nanocomposite sensor was precisely able to distinguish this virus from the influenza respiratory interference and other human coronaviruses (Figure 6).^73^

L-cysteine-derived CQD quenching behavior was used to determine the dopamine (DA) concentrations, an important hormone in neurotransmission pathways. The findings demonstrated that the fluorescence intensity of CQDs directly correlates to the concentration of DA in aqueous solutions.^66^ GQDs have also been successfully used as effective probes for sensitive and selective detection of DA, and upon the addition of DA in an aqueous solution, the initial strong blue fluorescence of the GQDs was effectively quenched. Transferring electrons from the photoexcited GQDs to DA–quinine, generated through DA oxidization by ambient O_2_ in an alkaline solution, causes quenching of the GQDs’ fluorescence. With a low detection limit, low cost, and high sensitivity, this developed sensing system proposes a potent tool for the quick and precise detection of DA in biological samples.^99^ A nano-biosensor was developed from metal chalcogenide embedded in an rGO-based electrochemical immunosensor and used to detect cTnI. This designed nano-biosensing platform exhibits a broad detection range with high sensitivity and a significantly lower detection limit, which is a potent biosensor for the detection of cardiovascular disease biomarkers.^74^ Similar biosensors fabricated of nitrogen-doped rGO exhibited a good ability to detect cardiac troponin I in concentrations of less than 1 pg/ml and offering great applicability in sensitive screening and routine monitoring of cTnI in clinical scales.^75^

**FIGURE 7:**
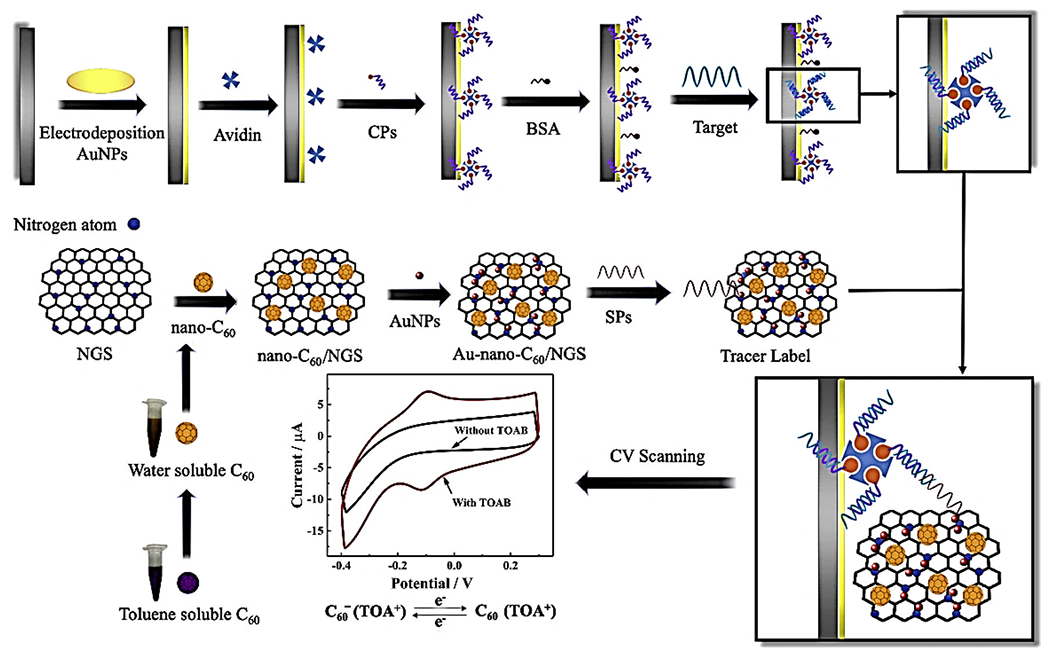
Stepwise process of fabricating the nanohybrid electrochemical DNA biosensor to detect *Mycobacterium tuberculosis* (MTB). Reproduced with permission.^76^ Copyright^©^ 2019 Elsevier B.V.

Fullerene (C60) is also used to design biosensors to detect various diseases, such as infectious diseases. To detect DNA fragments with *Mycobacterium tuberculosis* (MTB), a nanohybrid platform was designed using the gold nanoparticles decorated with fullerene nanoparticles/nitrogen-doped graphene nanosheet (Au-nano-C60/NGS). The fabricated electrochemical DNA biosensor provided a wide range for MTD detection with a low limit of detection. This nano-biosensor presents high efficiency in the detection of polymerase chain reaction (PCR) products of clinical samples (Figure 7).^76^

## 4 | THERAPEUTIC APPLICATIONS

The features of CNMs make them the most efficient and multifunctional candidates for various biomedical applications. Furthermore, the unique structure of CNMs with enhanced surface area helps to control their dimensions, which offers several superior prospective advantages as an advanced nanomaterial group.^100^ Over the past few decades, biomedical research has taken advantage of CNTs because of their exclusive characteristics. CNTs propose great perspectives for biomedical nano-devices because of their mechanical, chemical, thermal, and electrical features.^100^ These are now being used for therapeutic purposes and hold great interest in targeted drug delivery methods because of their simple transportation system through cell membranes and controlled drug delivery of several active pharmaceutical ingredients.^101^ This section will review some novel and important therapeutic applications of CNMs in treating cancer, brain disorders, and cardiovascular diseases (CVDs). Table 2. summarizes the CNMs in therapeutic applications.

### 4.1 | Cancer therapy

The cancer incidence rate has drastically risen and is the leading cause of death worldwide after cardiovascular disease. Environmental issues, such as urbanization, smoking, air pollution, changing dietary habits, and improved wealth associated with higher medical services quality, have been considered the main factors responsible for this phenomenon.^129^ Current standards of care comprise precise cancer staging with the techniques such as chemotherapy, radiotherapy, and/or surgical resection. Chemotherapy and radiotherapy are standard therapies with the most adverse effects that are non-specific methods and can rapidly target any dividing cells irrespective of whether or not they are tumorous.^130^

The appropriate size, charge, surface area, chemical composition, aggregation and/or agglomeration, and solubility of CNMs can highly affect their interactions with biomolecules and cells and make them promising candidates for developing new generations of anticancer systems.^4,131^

#### 4.1.1 | Anticancer drug delivery

CNTs, owing to their exceptional physicochemical properties, have been considered a general tool in cancer therapy.^132^ They are studied as one of the most promising nanomaterials capable of delivering drugs to cancerous cells and ideal candidates for lymphatic-targeted chemotherapy and gene therapy. Various types of functionalized CNTs (f-CNTs) possess the ability to be taken up by a broad range of cells and can intracellularly transit through different cellular barriers.^133^ Recently, various research groups have evolved many strategies to develop small carrier molecules such as chemotherapeutic cancer drugs, and load them on CNTs via either covalent conjugation or non-covalent adsorption.^134^ In the field of cancer therapy, the delivery rate of radionuclides or anticancer drugs via CNTs depends on two complementary approaches. The first strategy is to make non-covalent links between the nanotubes and the drug, the radionuclide alone or drug-polymer complex, and the second technique is based on forming bonds between compounds and tubes using a more stable covalent bond.^135^

Palbociclib (PLB), an anticancer drug, was absorbed by functionalized SWCNT (f-SWCNT) and functionalized single-walled silicon NT (f-SWSiNT) in vacuum and water. Based on the adsorption behavior and long-distance bonds, the PLB drug delivery rate was higher in f-SWSiNT than in SWCNT. The better performance of f-SWSiNT was attributed to its interaction strength and electronic sensitivity, making it a suitable candidate for drug delivery devices.^102^ Anticancer drugs can be delivered by covalent f-SWNT. The f-CNTs were combined with methotrexate (MTX) molecules, a well-known and potent anticancer drug. However, MTX lacks high bioavailability and low toxicity; therefore, improving its bioavailability and targeted delivery is highly advisable. A pH-sensitive hybrid system was developed using MWCNT coated with chitosan (CS) as a carrier for the target delivery of MTX into H1299 lung cancer cells. The nanohybrid showed high biocompatibility and affinity for MTX and facilitated sustainable pH-responsive drug delivery.^103^

In a research study, three different breast cancer xenograft mouse (SCID/Beige) models were treated with cisplatin (CDDP) encapsulated with ultra-short SWCNT capsules (CDDP@US-tube) materials to evaluate their biodistribution and therapeutic efficacy in vivo. This nanoplatform was found to have greater efficacy in retaining tumor growth compared to free CDDP for both BCM-4272 patient-derived xenograft model and the MCF-7 cell line xenograft model. These findings confirm the potential of the US-tube platform to facilitate the delivery of encapsulated CDDP by enhancing the accumulation of the drug in cancer resistance cells.^104^

To take advantage of C60 in drug delivery, it was successfully conjugated to the DTX. The fabricated nano-constructure could improve the bioavailability of DTX by 4.2 times and reduce drug clearance by 50%. The developed system enhanced the controlled drug release and was compatible with erythrocytes. The approach offered desired benefits of enhanced cancer cell cytotoxicity and a better pharmacokinetic profile.^105^

A nanoplatform was designed using poly(ethyleneimine) (PEI) and fullerene to load DOX anticancer drugs. This complex (C60–PEI–DOX) was employed to facilitate the integration of chemotherapy and PDT in a single system and DOX was covalently conjugated onto C60–PEI. The biodistribution of this nano-complex was evaluated by injection of CdSe/ZnS QDs labeled conjugates (C60-PEI-DOX/Qds) into tumor-bearing mice. By using C60–PEI–DOX/Qds complex, a higher tumor targeting efficiency was achieved compared with Qds alone. The ability of C60–PEI–DOX nanoparticles to dual local specific chemotherapy and external PDT remarkably enhanced the therapeutic efficacy of the cancer therapy.^106^

Another promising strategy to improve cancer therapy is the development of co-delivery systems incorporating therapeutic protein/peptides with small-molecule drugs or macromolecular nuclear acids. GO nanocarriers can efficiently release their cargoes, tumor necrosis factor-related apoptosis-inducing ligand (TRAIL), and DOX to their specific sites of action separately, in a site-responsive manner. Further, tumors harvested from the post-treated mice revealed that TRAIL/DOX-fGO caused the most significant shrinkage of tumor size.^107^

#### 4.1.2 | PTT and PDT

Phototherapy, which includes PTT and PDT, has been established in the past decades as a substitute technique for traditional cancer treatments to remarkably reduce adverse effects and improve selectivity.^136^ PDT is a type of light therapy that operates with visible light, a PS, and molecular oxygen to kill cancer cells.^137^ Among conventional therapies, PDT is non-invasive and selectively cytotoxic to malignant cells, which has been developed with great potential in treating cancer metastasis and can significantly improve the quality of life and prolong the cancer survival rate.^138^ In this therapeutic strategy, the NIR laser photoabsorbers are employed to make heat for the thermal ablation of cancer cells upon NIR laser irradiation.^139^

**FIGURE 8:**
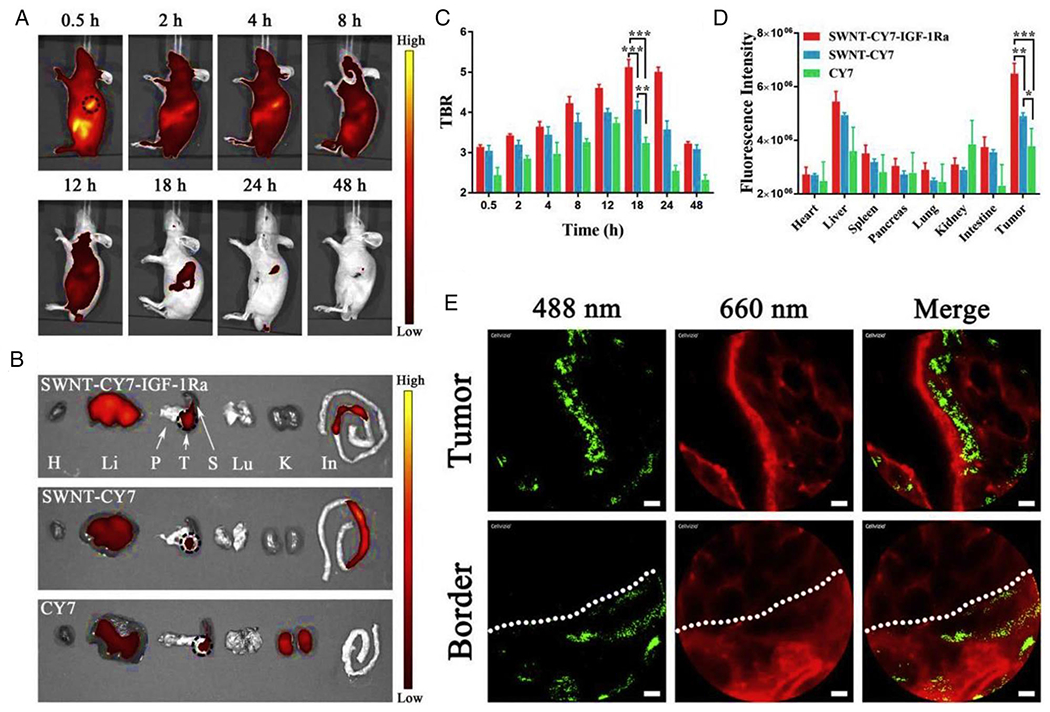
Biodistribution of dye-conjugated single-walled carbon nanotubes (SWCNTs) for photothermal therapy (PTT) of cancer. (A) In vivo continuous observations (48 h) of mice administered SWNT-CY7-IGF-1Ra via the tail vein. The black dotted circle represents the location of the pancreatic carcinoma in situ. (B) Ex vivo imaging of tumor and major organs. H: heart. Li: liver. P: pancreas. T: tumor. S: spleen. Lu: lung. K: kidney. In: intestine. (C) Comparison of tumor-to-background ratio (TBR) profiles of the nanoprobes. The peak was 18 h post-injection, implying an optimal experimental window. (D) Fluorescence intensity of different tissues. Data represent the mean ± SD of triplicate experiments. (E) The accumulation of as-prepared nanotubes along the tumor blood vessels and at the normal-tumor tissue junction at 18 h post-injection. The as-prepared nanotubes appear as green fluorescent dots at 488 nm, and the blood vessels are shown as red fluorescent regions at 660 nm. The scale bar is 20 *μ*m *p < 0.05, **p < 0.01, ***p < 0.001. Reproduced with permissions.^108^ Copyright^©^ 2019 Elsevier Ltd.

A specific type of dye-conjugated SWCNTs bound with targeting antibodies (IGF-1R) has been designed for PTT of orthotopic pancreatic cancer. This nanoprobe could specifically target pancreatic tumors for performing dyes imaging-guided cytotoxic PTT. The major restrictions in PPT techniques using metal NPs or organic dyes are low light-to-heat conversion efficiency and non-specific tumor targeting. This modified nanoplatform could overcome these barriers through the optical image-guided laser irradiation method, providing precise and excellent therapeutic effects with minimal adverse effects^108^ (Figure 8).

MWCNTs pre-loaded with DOX and CpG drugs are used as a powerful therapeutic complex for photothermal ablation. This complex can effectively facilitate the uptake of CpG by bone marrow-derived dendritic cells (BMDCs) and the maturation of BMDCs. Moreover, injection of this compound into the tumor site with subsequent NIR irradiation caused a deceleration of tumor growth rate in melanoma-bearing mice.^109^ Evaluation of PTT efficacy combined with MWNTs for bone metastasis of breast cancer demonstrated that MWNTs plus NIR laser irradiation induced significantly greater tumor growth suppression. Compared with treatment with either MWNTs injection or NIR irradiation alone, performing PTT and MWNTs injections remarkably raised the local temperature at the bone metastatic foci, reduced the mice tumor size, and protected the bone from cancer-induced damage.^110^

As another potent agent for PTT, graphene has started to threaten the dominance of CNTs in terms of greater efficiency of PTT, higher retention in tumors, and lower production costs. The graphene NPs exhibit some advantageous features over CNTs, for instance, inducing higher apoptotic/necrotic rates associated with triggering oxidative stress and mitochondrial membrane depolarization in cancerous cells. The photothermal anticancer efficiency of graphene nanoparticles in human glioma cell lines was greater than CNTs.^140–141^

Graphene-based nanomaterials have great potential for use as prognostic, diagnostic, and therapeutic agents in cancer photodynamic and photothermal therapies.^142^ The synthesized carboxylated photoluminescent graphene nanodots (cGdots) were excited at 655 nm, and then laser irradiation was applied by NIR (wavelength 670 nm) sensitizer. Upon excitation and laser irradiation, the electrons of the cGdots start to vibrate and form electron clouds that cause generating sufficient heat (>50°C) to effectively kill the cancer cells through thermal ablation.^111^ However, the poor solubility of graphene in aqueous solutions can limit their biomedical applications. GO, the oxidized form of graphene due to its enhanced water solubility, high surface area, and biocompatibility, has more advantages in biomedical applications. In particular, owing to their photothermal activity and high NIR intrinsic absorbance, they have aroused much more attention.^143^

Fullerene C60 has also been used as an efficient PS for PDT.^144^ It can generate ROS upon radiation, which can react with biological molecules such as unsaturated lipids, proteins, and nucleic acids and causes cell death through oxidative stress. However, its poor absorption in the NIR region of the spectrum makes C60 application for PDT strongly limited. Conjugation of C60 with a light absorber can fix this issue, and GO is a good candidate for this purpose. A hybrid of C60-GO enables dual PTT, and PDT induced by NIR light and exhibits high performance in inhibiting cancer cells compared to both individuals.^136^

Moreover, NIR light-harvesting fullerene-based NPs (DAF NPs) were employed for PA imaging-guided synergetic tumor photothermal and PDT. DAF NPs were injected into the xenograft human cervical carcinoma (HeLa) tumor mouse model, and 6 h after injection, they were accumulated in tumors and generated PA signals around the tumor. The ROS and heat generation efficacy was greater in DAF NPs than in fullerene and antenna NPs (DA NPs) alone.^112^

CQDs with a large surface area and available *π* electrons enable the immobilization of many chemical substances and offer several biomedical applications such as biomolecule sensing, drug delivery, PDT, and PTT.^145^ CQDs synthesized from polythiophene benzoic acid absorbs light in the 400–700 nm range and emit bright fluorescence in the red region. These carbon dots (CDs) can be used for both photodynamic and photothermal applications, and they exhibit a singlet oxygen-generating efficiency of 27% under 635 nm laser irradiation and high photothermal conversion efficiency of 36.2%.^113^ CQDs with unique up-conversion PL can serve as energy donors, which is beneficial for inducing PDT under NIR light and promoting tissue penetration. Loading DOX on fluorescence CQDs conjugated with folic acid as targeting ligand and riboflavin as PS improves NIR absorption and photothermal conversion efficiency, and controls intracellularly drug delivery.^114^ Hybridization of CQDs with silver/gold doped nanocomposite upon induction by NIR laser (808 nm, 2 W/cm^2^) led to death in cancerous HeLa cells. This CQD-NP complex could also improve targeting at the tumor site, which was proved by the blackening and reduction of the tumor sizes of treated mice.^115^

CNMs are also used as chemodynamic agents and have been reported to possess peroxidase activity similar to nanoenzymes activity. They can catalyze the decomposition of H_2_O_2_ into ^·^OH and therefore, they can be good candidates for chemodynamic cancer therapy.^146^ N-doped GO NPs with high NIR absorption and peroxidase-like activities were synthesized and employed for dual photothermal and chemodynamic purposes.^147^ Fe-N codoped carbon nanoparticles were used in tumor therapy and induced a Fe-based Fenton-like reaction to generate ^·^OH and O_2_ for chemodynamic therapy (CDT). This modified nanoplatform exhibited excellent potential in simultaneous CDT/PDT/PTT treatment of tumors.^148^ CNMs are also known as nanoenzyme and can induce different enzyme activities such as oxidase, peroxidase, superoxide dismutase, and catalase enzymes. In an effort to assess the anti-cancer potential of these materials, CNPs derived from beet were able to bind with a phosphate group and DNA bases and induced exhibit super high phosphatase nanozyme activity abrogating the CNPs-induced colony formation in anchorage-independent cancer cell growth.^149^

### 4.2 | Brain disorder therapy

**FIGURE 9:**
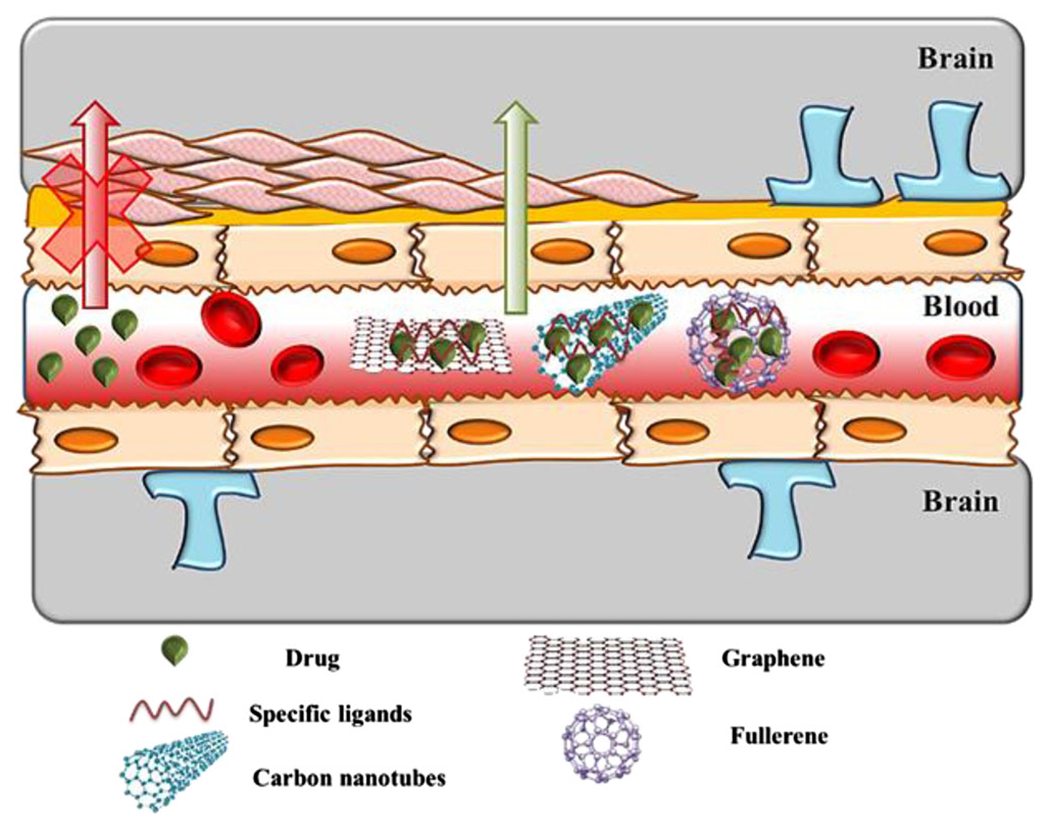
Proposed schematic diagram of carbon nanomaterial-based drug delivery to the brain.

Neurodegenerative diseases (ND) and brain tumors are major pathological defects affecting the brain. The Neuron network is very complicated, and to maintain the functionality of all the 86 billion neurons in the human brain, they need an adequate blood supply, provided through a vast, well-organized vascular network. Several cell types work specifically to conduct the blood into the cerebral blood flow and maintain blood-brain barrier (BBB) integrity.^150^ The primary requirement for the active absorption of drugs or active pharmaceutical ingredients (APIs) is to reach the absorbing barrier before reaching the BBB. Also, to ensure a controlled release profile, adequate absorption of APIs is required as they should pass through various barriers (Figure 9).

CNM family can be employed as highly efficient nanocarriers for drug delivery into the brain. Besides drug delivery applications, they have neuroregenerative activity and can also be used as central nervous system (therapeutic agents.^151^ Functional nanoparticles used in ND treatments are often fabricated using encapsulating active components into nanocarriers. These nanocarrier complexes provide prolonged drug circulation, protect the drug from degradation and elimination, enhance BBB accessibility, lessen cytotoxicity, and sometimes can actively reach the brain cells to perform more selective treatments.^152^

CNTs have much innovative potential for applications in neuro biomedicine, and one of the main applications in neuroscience is target drug delivery to treat ND. Single-walled carboxylated CNTs (SWCNT–COOH) have been successfully used as neuro-drug carriers to deliver levodopa (LD), the anti-Parkinson drug. The complex of SWCNT-COOH with LD was able to slow, sustainable release of the drug over a period of 20 h.^116^ Functionalized MWCNTs nanosystems caused an improved ability to penetrate the BBB, and it demonstrated high-efficient anti-tumor activity against orthotopic glioma. Therefore, developing such functionalized MWCNTs provides a novel technique for discovering next-generation nanodrugs to precisely treat orthotopic brain tumors.^117^

Misfolding and aggregation of amyloidogenic proteins is a key factors in Alzheimer’s disease (AD) occurrence. Therefore, one of the effective strategies to treat AD is the inhibition of amyloidogenic protein aggregation. To study the inhibitory effects of CNTs on the amyloid *β* fibrillation process, SWCNT-COOH was developed based on the hydrophobic binding-electrostatic repulsion mechanism. The SWCNT-COOH could attenuate A*β* aggregation and induce an anti-A*β* neuroprotective effect. The dissociation of A*β* stability and internal structure by SWCNT-COOH was demonstrated using molecular dynamics simulations.^118^

The impact of CQDs sourced from Na–citrate was investigated on the amyloid-fibril-forming in hen-egg white lysozyme (HEWL), as a model amyloidogenic protein trajectory. The results revealed CQDs’ inhibitory effects on converting monomeric and oligomeric HEWL intermediates into mature fibrils. Moreover, and interestingly, they can dissociate oligomeric proteins into monomeric units and induce the diffusion of mature HEWL fibrils, suggesting their therapeutic intervention.^119^ Lack of DA due to the death of dopaminergic neurons in the brain is one of the main causes of Parkinson’s disease. One of the currently available treatments is the administration of L-DA in the early stages of the disease, which can cross the BBB and convert L-dopa to DA. DA loaded on CS was conjugated with CDs and made a nanocomposite matrix of DA@CS/CDs. The drug-encapsulated nanocomposite exhibited sustained and controlled release of DA, which ensures that more amount of the drug will be utilized effectively.^120^

**FIGURE 10:**
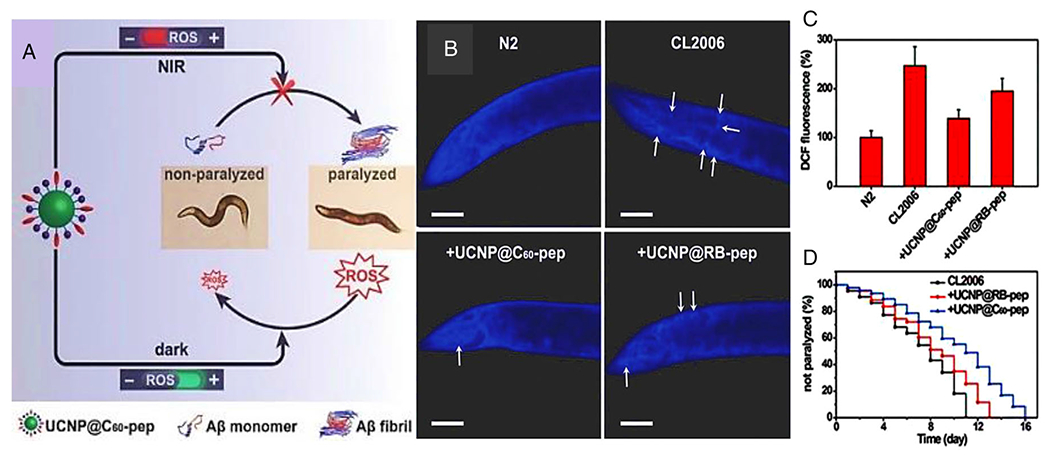
A carbon nanotube (CNT)-based theranostic nanoplatform for Alzheimer’s disease (AD) treatment (A) Upconversion nanoparticle (UCNP@C60-pep) inhibited A*β* aggregation in vivo and attenuated the oxidative stress to prolong the lifespan of Caenorhabditis elegans (CL2006) strain. UCNP@C60-pep produced reactive oxygen species (ROS) under near-infrared (NIR) to disturb A*β* aggregation and scavenged the overproduced ROS in the dark. As a result, UCNP@C60-pep could ameliorate A*β*-triggered paralysis in CL2006 worms. (B) Representative ThS-staining images of N2 and UCNPs-treated CL2006 strains on the sixth day after the addition of the UCNPs. The wild-type N2 strain was used as the control. Scale bars are 40 *μ*m. (C) The ROS level of worms was detected by DCF fluorescence on the sixth day. (D) Survival curves of UCNPs-treated CL2006 strain. Strains were classified as paralyzed if they failed to respond to a touch-provoked movement. Reproduced with permission.^59^ Copyright^©^ 2018 WILEY-VCH Verlag GmbH & Co. KGaA, Weinheim.

To explore the novel treatment of Alzheimer’s disease, an aqueous colloid solution of C60 was used to treat degraded hippocampal pyramidal cells with the deposition of *β*-amyloid. After treatment with a C60-containing solution, cells showed reduced amyloid plaques and prevented disruptions in the protein synthesis pathway and neurodegeneration. It was concluded that functionalized C60 might be an excellent anti-amyloid therapeutic drug because of its antioxidant and anti-aggregative properties.^121^ A nanoplatform made of C60 and amyloid-*β* (A*β*)-target peptide KLVFF24 was conjugated to an upconversion nanoparticle (UCNP@C60-pep) and employed as the A*β*-targeted theranostic system for AD treatment. This nanosystem exhibited neuroprotection to AD model *Caenorhabditis elegans* through ROS production and inducing the A*β* photooxygenation, which can hinder A*β* aggregation (Figure 10).^59^

### 4.3 | CVD therapy

CVDs are considered the leading cause of death worldwide. Atherosclerosis is the main cause of CVDs, leading to ischemic heart disease, mesenteric ischemia, renovascular disease, peripheral vascular disease, and cerebrovascular disease.^27^ In traditional techniques, the damaged section was treated for thrombosis, and the artificial pace-maker was placed for arrhythmia. However, they were slightly suitable therapies, but not many patients were satisfied because of making discomfort for their life-time. The necessity of overcoming these issues brought the role of nanotechnology into the limelight. They can easily be penetrated inside tight junctions due to their small size. By having the tunable shape and the increased surface area, they can provide more comprehensive options for surface modification and binding.^153^ These unique properties make them exceptional options compared to other biomaterials for cardiovascular theranostic. Here, some of the features of the most typically employed CNMs and their derivatives are discussed with respect to their potential applications in cardiovascular theranostic.^27^

**FIGURE 11:**
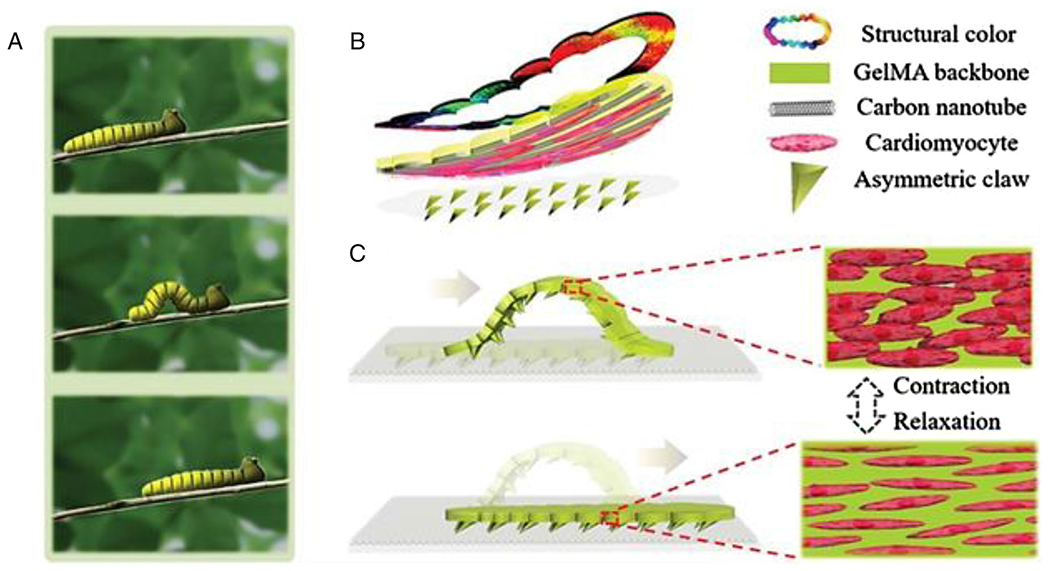
Schemes of the caterpillar-like soft robot. (A) The crawling mechanism of a caterpillar in nature. (B) Schematic diagram of the caterpillar-like soft robot composed of three layers. (C) The crawling process of the soft robot is driven by cardiomyocytes. Reproduced with permission.^122^ Copyright^©^ 2019 WILEY-VCH Verlag GmbH & Co. KGaA, Weinheim.

There are some reports indicating the cytocompatibility of CNTs with cardiomyocytes and neurons and their potential for myocardial tissue engineering applications.^123^ Mechanical coupling of cardiomyocytes (heart cells) to each other and the formation of elongated and aligned cell bundles that create an anisotropic syncytium can be facilitated by the extracellular matrix of muscle tissue, like the heart. Recently a novel biological soft robot has been designed using a CNTs-induced myocardial tissue layer and a color probe that simulates the crawling of snakes and caterpillars (Figure 11). Cardiomyocytes’ arrangement can be regulated using the CNTs layer by improving the beating capability and the contraction performance. The motion status was dynamically and precisely visualized and monitored using the color probe in this biological soft robot.^122^

**FIGURE 12:**
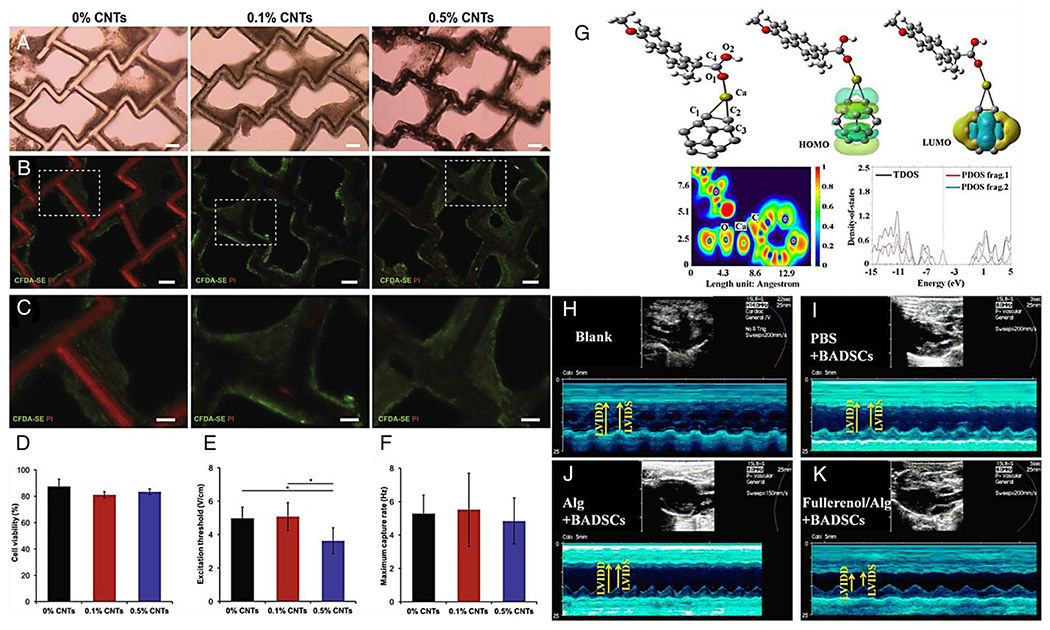
Composite scaffolds in cardiac tissue engineering. (A–C) Images of rat cardiac tissue (A) Brightfield images of polymers seeded with rat CMs (Scale bars: 100 *μ*m). (B) Tissues viability images using live/dead staining assay (Live cells: green, dead cells: red) (Scale bars: 100 *μ*m). (C) High magnification of wrapping of viable cells around scaffold struts (Scale bars: 50 *μ*m). (D) Cell viability quantification of live-dead images. (E) Comparison of excitation threshold (F) and maximum capture rate (*p < 0.05). (G) Adsorption configuration, Frontier molecular orbital (FMO), electron localization function (ELF), and DOS plots of NPX/Ca(C20) adsorption complex. (H,K) Assessment of in vivo cardiac functions of infarcted hearts after treatment with hydrogel combined with BADSCs. Representative echocardiography images of MI model rat in (H) blank group, (I) phosphate-buffered saline (PBS)+BADSCs group, (J) Alg+BADSCs group, and (K) fullerenol/Alg+BADSCs group. Reproduced with permissions from references.^124,127–128^ Copyright^©^ 2016 Acta Materialia Inc. Published by Elsevier Ltd., ^©^ 2022 Elsevier B.V., and ^©^ 2017, American Chemical Society.

The application of CNTs to cultured cardiomyocytes enhances their viability and proliferation and accelerates their maturation. Moreover, the endothelial cells in blood vessels can be supported by these systems for supplying oxygen to the heart muscle.^154^ The many ways that CNTs can alter material characteristics include the addition of CNTs to biodegradable poly(lactic-co-glycolic-acid) (PLGA) polymers and assessing their conductivity. For CVD applications, Ahadian et al. used a polyester matrix and CNTs to prepare the polymeric nanomaterials with improved electrical conductivity, stability, and stimulation of cell-cell coupling. Hybrid polymeric scaffolds containing 0.5 wt% CNTs compared to pure polymeric scaffolds could improve the maturation of cardiac tissues fabricated on them (Figure 12A–F).^124^ CNTs can improve the conductivity of scaffolds, increase fiber diameter, and promote cellular migration into the scaffolds. In a recent report, the electrospun CNT-based scaffolds have been fabricated using sandwich and dual deposition methods. By incorporating CNTs into the scaffolds during the electrospinning part of the process, their electrical conductivity and cytocompatibility were significantly improved, which makes them potential candidates for developing novel cardiac tissue-engineered constructs.^123^

Carbon nanomaterials such as CQDs, due to their high fluorescence properties, are potent materials for detecting cardiac markers. Myoglobin (Mb) is one of the most reliable markers for predicting future cardiovascular events and/or diagnosing patients with acute coronary syndrome. CQDs synthesized from L-glutamic acid are synthesized and functionalized with generated anti-Mb-Aptamer and can be employed as a sensitive assay platform for the selective detection of the cardiac marker Mb.^125^ Occlusion of blood vessels is a serious concern arising as a consequence of atherosclerosis, and therapeutic angiogenesis is a strategy to develop new blood vessels from the pre-existing ones. Vascular endothelial growth factor (VEGF) is a well-studied positive regulator which exerts therapeutic benefits by inducing long-lasting angiogenesis in ischemic cardiac tissue. Tracking VEGF’s transfer to ischemic tissues reveals its biodistribution and delivery to address the therapeutic angiogenesis and tissue engineering requirements.^155^ A microfluidic microgel containing VEGF-conjugated fluorescent C-dots (VEGF–Cdots) has been developed for imaging monitoring of VEGF delivery to ischemic muscles. The VEGF–Cdots were released at a sustainable rate and with high bioactivity. The microgels exhibited a strong fluorescent signal and great angiogenesis potential, resulting in improved therapeutic angiogenesis of ischemic muscles.^126^

Fullerene is a well-known powerful free radical sponge that can deactivate free radicals such as hydroxyl and superoxide anion both in vivo and in vitro. Overproduction of ROS and induction of oxidative stress are one of the main causes of ischemia and CVDs. Fullerene C60 presents great potential in cardiovascular disease therapy by reducing ROS production, triggering angiogenesis, and accelerating cardiac functional recovery.^128^ Inhibition of proinflammatory cytokines is a functional strategy to reduce cardiovascular risk in rheumatoid arthritis disease. Sulfasalazine, curcumin, and naproxen as anti-inflammatory drugs are functionalized with C20 fullerenes and exhibit higher inhibitory activity than pure drugs. Moreover, C20 fullerene improves the physicochemical features of the drugs such as absorption, distribution, metabolism, and excretion, and, therefore, can be considered an excellent material for delivery (Figure 12G).^127^ Fullerenol nanoparticles, as fullerene derivatives, were introduced into alginate (Alg) hydrogel and their impact on brown adipose-derived stem cells (BADSCs) and were able to suppress the oxidative stress damage of BADSCs. This implantation strategy significantly reduced myocardial infarction ROS level, improved the survival of implanted BADSCs, and triggered angiogenesis, which in turn expedited cardiac functional recovery (Figure 12H–K).^128^

## 5 | CONCLUSION AND PERSPECTIVES

This review presents a summary and discussion of the recent advances in CNMs, particularly their application in biomedical fields. The CNMs have many unique features: their small size, ultrahigh surface area, excellent mechanical strength, low toxicity, biocompatibility, and electrical, thermal, and optical properties, which make them potent candidates for theranostic applications. These multifunctional nanostructures offer various superior promised advantages compared to the other nanomaterials, and therefore, recently, much research aimed at designing diagnosis and therapeutic devices using CNMs. There are currently prominent advances in developing CNMs-based biomedical techniques, including cancer therapy, drug delivery, bioimaging, and biosensing. These CNMs have been considered in the pre-clinical and clinical stages to investigate their potential as diagnostics probes. Here, we have also discussed the potential application of various CNMs in cancer therapy through drug delivery systems, PTT and PDT, and treating brain disorders and CVDs. In particular, the flexibility of these nanomaterials for modification provides the opportunity for their combination with other functional nanomaterials to improve their efficiency and applicability for theranostic systems. These nanoplatforms can easily combine the exceptional CNMs properties into a unique system that presents multifunctions to detect, image, and treat different diseases. The multifunctionalization of CNMs will provide novel perspectives in biomedical applications and integrate these materials into different nanosystems.

However, the progress of CNMs-based diagnostic and therapeutic and their clinical translation area is still in the infancy. There are critical challenges to establishing CNM-based techniques in clinical scales which need to be addressed before any real clinical application. The major efforts on CNMs-based diagnostic technologies are not well developed and are still at the proof of concept stage or pilot scale. There are some critical challenges in applying these nanomaterials for clinical purposes that need to be addressed. First, although there has been a large amount of research aimed at investigating the safety of CNMs for clinical applications, the toxicity of CNs is still a major concern. The research on CNMs degradation is mainly focused on oxidized CNMs which have desirable biocompatibility and dispersibility. However, the degradation process of CNMs depends on the synthesis methods, and different degrees of aggregation during preparation can affect their interactions with the enzymes and their degradability, which in turn increases the risk of their toxicity in living organisms. Although there are many reports on the safety and nontoxicity of CNMs for biomedical applications, there is still no in-depth information available covering all these concerns. Therefore, more advanced research is required to validate their long-term biosafety as opposed to their therapeutic potential and, eventually, their clinical application.

Second, CNMs-based devices applied for diagnosis techniques can only provide clinicians with references for further trials and cannot be considered as a direct basis for diagnosis. One of the main issues in the application of these materials for the detection of bio-analytes or biomarkers in specific diseases such as CVD is their sensitivity and specificity. Therefore, the accurate detection of analytes in human samples still needs more advanced established techniques like quantitative real-time-PCR. Developing more sensitive CNM-based devices with ideal analytical detection limits can considerably improve their efficiency and accuracy for biomedical applications.

Moreover, the CNM-based devices developed to diagnose and treat diseases suffer from a lack of stability. The oxidation process during the synthesis of the CNMs causes instability of the final products because of their different surface functional groups. The heterogeneity in the batch production of these materials creates uncertainties for their application for theranostic techniques. Overall, the CNMs exhibited the unique potential to be employed for the non-invasive theranostic systems and bring new hope for advancing the diagnosis sector for a wide range of diseases such as cancer, brain disorders, and CVDs in the near future.

## Figures and Tables

**TABLE 1:** Diagnosis applications of carbon-based nanomaterials.

Techniques	CNMs-compound	Site of action	Application	Results	Ref.
**Bioimaging**					

Magnetic resonance imaging (MRI)	CNT-gadolinium-diethylenetriamine pentaacetic acid (Gd-DTPA)	MCF7 cells	Deliver Gd-DTPA	Improving target tumor site	^52^
	GNTs	BMDMSC cells	MRI agent	Labeling cells for MRI and phantom imaging	^53^
	MWCNTs-Iron	Mice leg	MRI agent	Reduced iron toxicity/Localization in tumors	^54^
	CNT fibers	Brain	MRI agent	High interfacial-electrochemical properties/Improve brain targeting	^55^
	Fe_3_O_4_@CQDs-SWNTs	A549, HeLa cells	Multifunctional MRI probe	Drug loading carrier/near-infrared (NIR) photothermal heater and ROS generator	^56^
	f-CQDs (BNSCQDs)	HepG2, PC3, and 3T3 cells	MRI agent	Improve relaxivity/great fluorescence	^57^
	Gd_3_-NPs	A*β*-PC12 cells	MRI agent	High-resolution contrast between tumor and healthy tissues	^58^
	C60	Neural PC12-A*β* cells	AD diagnosis	Visualize and colocalize in neural cells	^59^
	MFG-SiNc4	HeLa cells	-	Improve relaxivity of gadofullerene	^60^
	GO-IONP	Regional lymph nodes (RNL)	Nanotheranostic agent	Detect cancer metastasis region	^61^

Fluorescence Imaging	DNA-encapsulated SWNTs	Liver, spleen, lung, kidney, and heart	-	Short and long-term biodistribution/long-retention in organs	^62^
	f-SWNTs @phospholipid polymers	Breast tumor	Photoluminescence probe	Intrinsic NIR/video rate tumor imaging/precise tumor site detection	^63^
	N-doped CQDs	*C. elegans* (in vivo model)	Multicolor imaging	-	^64^
	C-dots	HeLa tumor-bearing mice	Fluorescent probe	High permeability and retention effect	^65^
	L-cysteine derived CQDs	Gastric carcinoma cells	Fluorescent probe	Excellent luminescence properties	^66^
	Sulforaphane-conjugated C-dots	A549	Fluorescent probe	Target cancer cells/high compatibility and optical stability	^67^
	GO-polyethyleneglycol (PEG)-folate complex	Melanoma cancer cells	-	Localization of melanoma cancer cells	^68^

**Biosensor**	SWCNTs	PBS and lysed blood	Peroxide-like sensor	Detect rare number of CTCs	^69^
	GQD and SWCNTs	-	Enzyme-free electrochemical immunosensor	Detect carcinoembryonic antigen (CEA)	^70^
	Carboxylated MWCNTs	-	Nano-sensor	Detection of cardiac Troponin I	^71^
	Au-NPs-fluorine-doped tinonid @ SWCNTs	-	Nano-genosensor	Detect miR-21 in prostate cancer	^72^
	CNTs/WO3	-	Electrodes of the sensor	Detect SARS-CoV-2 virus particles	^73^
	L-cysteine-derived CQDs/GQDs	-	Nano-bio-sensor	Detect the dopamine hormone concentrations	^66^
	rGO	-	Electrochemical immunosensor	Detect cTnI in low concentrations	^74^
	N-doped rGO	-	Nano-sensor	Detect cTnI in low concentrations	^75^
	Au-NPs-C60	-	Electrochemical DNA biosensor	Mycobacterium tuberculosis direct (MTD) detection with a low limit of detection	^76^

**TABLE 2:** Therapeutic applications of carbon-based nanomaterials.

Technique	CNMs-compound	Site of action	Application	Results	Ref.
**Cancer therapy**					

Anticancer drug delivery	f-SWCNT and f-SWSiNT	-	Delivery of the palbociclib anticancer drug	-	^102^
	f-MWCNT-methotrexate (MTX)@Chitosan	H1299	MTX drug delivery	Facilitate sustainable target delivery	^103^
	CDDP@US-tube	MCF-7, BCM-4272	Anticancer drug delivery	Enhancing drug retention in cancer resistance cells	^104^
	C60-docetaxel (DTX)	MCF-7, MDA-MB231	DTX drug delivery	Enhance drug bioavailability and cancer cell cytotoxicity/Reduce drug clearance	^105^
	PEI-C60-DOX (labeled with QDs)	B16-F10	Dual chemotherapy and photodynamic therapy	High tumor targeting efficiency	^106^
	GO	A549, LoVo cells	Co-delivery of TRAIL and DOX	Significant shrinkage of tumor size	^107^

Photothermal and photodynamic therapy	dye-conjugated SWCNTs-(IGF-1R)	Pancreatic tumor	Photothermal therapy (PTT)	Specific target for dyes imaging-guided cytotoxic PTT	^108^
	MWCNTs-DOX-CpG	BMDCs	Photodynamic therapy (PDT)	Facilitate the uptake of CpG, maturation of BMDCs/Deceleration of tumor growth rate	^109^
	MWCNTs	MCF7-MDA-231	PTT	Tumor growth suppression	^110^
	cGdots	MDA-MB231	PTT	Generate heat and kill the cancer cells	^111^
	DAF NPs (fullerene-based)	Xenograft HeLa tumor mouse	PTT	Generate PA signals/ROS and heat generation	^112^
	CQDs (synthesized from polythiophene benzoic acid)	HeLa cells/Liver, lung, and kidney	PTT	Improve singlet oxygen generating	^113^
	DOX-CQDs-folic acid	HeLa cells	PTT	Improve NIR absorption	^114^
	CQDs-silver/gold doped	Human cervical carcinoma (HeLa) cells	PDT	Kill cancerous cells/Blackening and tumor sizes reduction	^115^

**Brain disorder therapy**	SWCNT–COOH	PC12	Levodopa drug delivery	Slow, sustainable drug delivery and release	^116^
	f-MWCNTs	orthotopic glioblastoma	Anti-tumor	Improve BBB penetration	^117^
	SWCNT-COOH	-	Neuroprotection	Attenuate A*β* aggregation/Dissociation of A*β* stability/	^118^
	CQDs sourced from Na–citrate	-	-	Inhibition of converting HEWL intermediates into mature fibrils	^119^
	Dopamine@CS/CDs	IC21, SH-SY5Y	Dopamine delivery	Sustainable and controlled release of dopamine	^120^
	C60	-	Neurodegeneration inhibitor	Reduce amyloid plaques/Anti-aggregative properties	^121^
	Upconversion nanoparticle (UCNP)@C60-pep	*C. elegans* model	Neuroprotection	Reduce ROS production/Prevent A*β* aggregation	^59^
**Cardiovascular disease therapy**	CNTs	-	Biological soft robot	Simulation of myocardial muscle movements	^122^
	CNTs	-	Cardiac tissue-engineering	Improve scaffold’s electrical conductivity and cytocompatibility	^123^
	Polymeric CNTs	Cardiac tissue	Cardiac tissue-engineering	Improve electrical conductivity and cardiac tissues maturation	^124^
	L-glutamic acid derived-CQDs-anti-Mb-Aptamer	-	Biomarker	Detection of the cardiac marker Mb	^125^
	Vascular endothelial growth factor (VEGF)@C-dots	Ischemic muscles	Drug delivery/Angiogenesis	Sustainable releasing of VEGF/Improve angiogenesis	^126^
	C20@(Sulfasalazine, curcumin, and naproxen)	-	Drug delivery	Improve drug absorption, distribution, metabolism, and excretion	^127^
	Fullerenol/Alginate	BADSCs	Angiogenesis	Reduce myocardial ROS/Expedite cardiac functional recovery	^128^
